# Formation and healing of pectoral fin bite marks in a female whale shark *Rhincodon typus*

**DOI:** 10.7717/peerj.21809

**Published:** 2026-10-23

**Authors:** Rui Matsumoto, Kiyomi Murakumo, Kei Miyamoto, Taketeru Tomita

**Affiliations:** 1 Zoology Laboratory, Okinawa Churashima Foundation Research Institute, Motobu, Okinawa, Japan; 2 Fish Husbandry Section, Okinawa Churaumi Aquarium, Motobu, Okinawa, Japan; 3 Veterinary Hospital, Okinawa Churashima Foundation, Motobu, Okinawa, Japan

**Keywords:** Whale shark, *Rhincodon typus*, Bite marks, Pectoral fin, Wound healing, Pigmentation, Female whale shark, Scar analysis, *Ex situ* observation, Elasmobranch

## Abstract

Biting behavior by males during mating has been widely documented in elasmobranchs; however, its characteristics in the whale shark, *Rhincodon typus*, remain poorly understood. In this study, we recorded a pectoral fin biting event by a male directed toward a female at Okinawa Churaumi Aquarium on May 12, 2020, using surveillance cameras. Subsequently, we monitored the healing process of the resulting scars through underwater observations and post-mortem tissue analyses over 13 months. The bite marks initially appeared as open wounds, which closed within approximately 4 weeks, and were followed by the regeneration of dermal denticles and progressive melanization that persisted for more than 13 months. Additionally, 523 bite marks (233 dorsal and 290 ventral) were digitized and analyzed using orientation-based analysis and Gaussian mixture model clustering. Three distinct clusters were identified among bite marks attributed to this single documented interaction event, suggesting that variation in jaw orientation or repositioning may have occurred during the event. In this individual, wound closure occurred within approximately 4 weeks, providing a preliminary reference for the temporal interpretation of recent injuries, while melanized scars remained externally visible for more than 13 months. These observations provide a preliminary framework for evaluating pectoral fin bite marks as potential indicators of male–female interaction history in whale sharks, although validation across additional individuals is required. These findings provide a foundation for future studies integrating behavioral observations and scar-based assessments in both *ex situ* and *in situ* contexts.

## Introduction

Courtship-associated biting behavior is widely documented across elasmobranchs, where males grasp the pectoral fins or flanks of females during reproductive interactions to maintain position for clasper insertion (Pratt & Carrier, 2001). These interactions frequently leave characteristic bite marks, which have been used as externally visible indicators of male–female encounters in both field and captive studies (Carrier, Pratt & Martin, 1994; Whitney, Pratt & Carrier, 2004). Beyond their behavioral significance, the spatial distribution and healing stages of such marks can provide insights into the timing, frequency, and intensity of male–female interactions, contributing to the identification of mating seasons and aggregation dynamics (Domeier & Nasby-Lucas, 2007; Marshall & Bennett, 2010; Chin, Mourier & Rummer, 2015). In some species, fresh bite marks have been associated with reproductive condition, such as ovulation or fertilization periods (Jensen et al., 2002), although such interpretations require careful validation. As a result, the study of bite marks represents a valuable, non-invasive approach for investigating otherwise cryptic reproductive processes in elasmobranchs, with important implications for conservation and population management.

The whale shark, *Rhincodon typus*, is the largest extant fish, reaching total lengths exceeding 13 m (Acuña-Marrero et al., 2014), and is widely distributed across tropical and temperate oceans (Stevens, 2007; Cochran et al., 2019). Despite extensive research on its distribution, migration, and aggregation ecology over the past two decades (Norman et al., 2017; Araujo et al., 2022), its reproductive biology remains poorly understood. This species is yolk sac viviparous and capable of producing large litters (Joung et al., 1996), yet key aspects such as mating grounds, mating frequency, and gestation period remain unknown (Reynolds et al., 2022). Direct observations of courtship or copulation are extremely rare (Perry et al., 2020; Barry et al., 2025), largely due to the species’ wide-ranging pelagic lifestyle and the offshore distribution of mature individuals (Hearn et al., 2021). To date, only a few fragmentary observations of interaction events, including pursuit and contact behaviors, have been reported, and no study has tracked the formation and healing of bite marks over time. Consequently, the role of such scars as indicators of male–female interactions in this species remains unclear.

*Ex situ* research conducted in aquaria and other controlled environments provides complementary opportunities to investigate behavioral processes that are often difficult to observe in free-ranging animals. This is particularly important for large, wide-ranging, or cryptic species. In elasmobranchs, captive studies have contributed substantially to our understanding of reproductive biology, including courtship behaviors, copulation mechanics, and embryonic development (Uchida, Toda & Kamei, 1990; Murakumo et al., 2020; Feldheim, Wyffels & Lyons, 2022). Observations under managed conditions have revealed behavioral sequences rarely documented in the wild, including prolonged pursuit and repeated fin biting in zebra sharks, *Stegostoma tigrinum*, and pursuit followed by pectoral-fin biting, positioning, and copulation in *Triaenodon obesus* (Watson & Janse, 2017; Santos et al., 2022). At the Okinawa Churaumi Aquarium, whale sharks have been maintained long-term, and a male individual has been confirmed to reach sexual maturity and exhibit reproductive behaviors (Matsumoto et al., 2019). Repeated contact behaviors toward an immature female have also been observed, providing a rare opportunity to document interactions that may result in bite marks.

In this study, we documented a pectoral fin biting event by a male whale shark and subsequently tracked the condition and healing trajectory of the resulting bite marks in a female over an extended period using surveillance observations. Based on this unique dataset, the objectives of this study were to (i) document the behavioral observations associated with the formation of pectoral fin bite marks, (ii) describe the long-term healing process of these scars, and (iii) quantitatively analyze the geometric characteristics of the bite marks. We further evaluate the extent to which such scars can function as indicators of male–female encounter history and timing, and discuss how these findings can contribute to *in situ* studies of whale shark reproductive ecology and inform conservation strategies for this endangered species (Pierce & Norman, 2016).

## Materials and Methods

### Experimental subjects

The female whale shark shown in Fig. S1A was an immature individual housed in the Kuroshio Sea tank (approximately 7,500 m^3^) at the Okinawa Churaumi Aquarium (Fig. 1A). The tank was supplied with natural seawater pumped from 300 m offshore at a depth of 20 m, and the water temperature varied seasonally, from approximately 20 °C in winter to 30 °C in the summer. The upper portion of the tank was covered with glass, allowing natural sunlight during the day, while artificial lighting was provided from 0700 to 1830 h. Outside these hours, illumination was minimized for safety monitoring.

**Figure 1:**
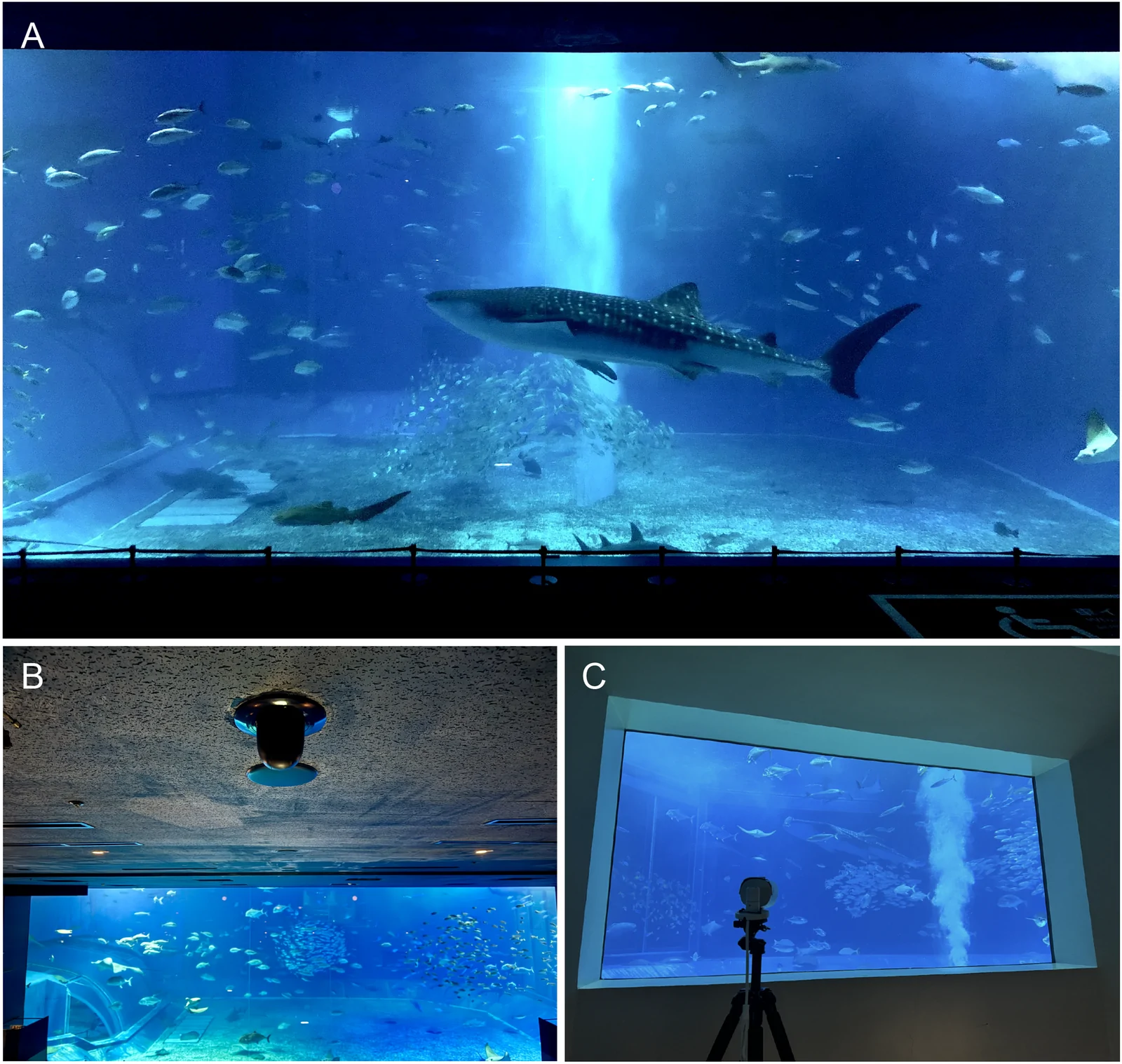
Kuroshio Sea tank at Okinawa Churaumi Aquarium used for housing whale sharks. (A) Main front viewing panel; (B) surveillance cameras mounted on the ceiling of the viewing gallery; and (C) supplementary monitoring camera installed at the opposite observation window.

The total length of the female was 8.0 m. Skeletal abnormalities, likely caused by external trauma before captivity, gradually worsened over time and ultimately contributed to her death in June 2021 (Okinawa Churaumi Aquarium Official website: https://churaumi.okinawa/en/topics/1623465430/). Necropsy confirmed that her reproductive organs were immature, indicating that she had not yet reached sexual maturity. During her captivity (4,824 days from April 2008), she was fed daily with 0.5–0.9% of body mass, which primarily consisted of euphausiids, mysids, and chopped fish. The same tank also housed a male whale shark measuring 8.8 m in length (Fig. S1B). This individual was confirmed to have reached sexual maturity in 2012 (Matsumoto et al., 2019) and was fed daily at 0.5–0.8% of its body mass, with the same diet composition as that of the female.

### Ethics statement

This study was conducted as part of routine animal health management and observational research activities at the Okinawa Churaumi Aquarium. All observations were non-invasive and non-interventional, and no physical contact, experimental manipulation, or additional procedures beyond standard husbandry and veterinary care were performed. Accordingly, approval from an institutional animal ethics committee was not required for this study. All animals were maintained in accordance with the Animal Welfare Guidelines of the Japanese Association of Zoos and Aquariums and the Animal Welfare and Behavioral Management Policy established by the Okinawa Churashima Foundation.

### Behavioral monitoring

The Kuroshio Sea tank housing the two whale sharks was continuously monitored using surveillance cameras (WV-SC588; Panasonic Corp., Kadoma, Osaka, Japan) installed in the observation area to support animal health management and facility safety (Objective i, Fig. 1B). Video footage was recorded for 24 h and subsequently reviewed to document male–female interaction behavior directed toward females by males. To compensate for the blind spots of the primary camera, an auxiliary camera (WTW-P27HT-64GB; Wireless Tsukamoto Co. Ltd., Tsu, Mie, Japan) was installed in front of the observation window opposite the main camera (Fig. 1C). This auxiliary camera also recorded continuously, and footage from both camera systems was integrated for subsequent behavioral analyses. All recorded footage was reviewed for biting events involving the female’s pectoral fins.

### Monitoring the healing process of bite marks

To document the healing process of pectoral fin bite marks inflicted on the female whale shark (Objective ii), underwater observations were conducted intermittently from May 12, 2020, until the death of the individual on June 17, 2021. Both dorsal and ventral surfaces of the pectoral fin were photographed using a digital action camera (GoPro Hero 9 Black™; GoPro Inc., San Mateo, CA, USA) and a digital camera (OLYMPUS STYLUS XZ-2; Olympus Corp., Hachioji, Tokyo, Japan). Following the death of the female, a necropsy was performed, and skin samples were collected from scarred regions on both the dorsal and ventral surfaces of the pectoral fin. The tissue samples were fixed in 10% buffered formalin. After fixation, the condition of the skin and regenerated dermal denticles were examined under a stereomicroscope (LEICA M205 C; Leica Microsystems, Shinjuku, Tokyo, Japan), and high-resolution images were obtained for documentation.

### Detailed analysis of bite marks

To address the study objective of quantitatively characterizing bite mark geometry (Objective iii), we conducted a detailed geometric analysis of individual bite marks. In addition, to support the interpretation of these patterns in relation to interaction processes, we explored whether spatial and directional structures may provide insights into repeated biting behavior within a single interaction event.

After necropsy, transparent sheets were overlaid on the dorsal and ventral surfaces of the pectoral fin, and fin outlines together with all visible bite marks were manually traced. To minimize subjective variation, all tracings were performed by the same observer. The traced sheets were digitized using a scanner (imageRUNNER ADVANCE C5540F; Canon Inc., Ota, Tokyo, Japan). Bite marks were then retraced as Bézier curves using Adobe Illustrator (Adobe Inc., San Jose, CA, USA) and saved in the scalable vector graphics (SVG) format. For alignment, ventral images were flipped horizontally based on fin outlines to allow direct comparison with dorsal surface.

From the SVG files, each <path> element corresponding to a bite mark was extracted using Python (version 3.11) and the svgpathtools library (version 1.7.1). Each curve was resampled into 100 equidistant points, enabling the geometry of individual scars to be treated as numerical data.

Principal component analysis (PCA) was applied as a dimensionality-reduction tool to extract the dominant orientation axis of each bite mark from complex curve geometries. The first principal component (PC1), representing the direction of maximum variance, was used as a proxy for the primary orientation of each scar. This approach allowed each bite mark to be summarized by a single interpretable parameter (orientation angle), facilitating comparison among scars while minimizing the influence of irregular shape variation.

To explore whether the distribution of bite mark orientations exhibited structured patterns rather than random variation, Gaussian mixture model (GMM) clustering was applied independently to the dorsal and ventral datasets using PC1-derived orientation angles as input variables. This analysis was conducted as a descriptive and exploratory procedure to identify potential grouping structures within the data, which may reflect repeated biting positions or consistent jaw orientations during interactions. The optimal number of clusters was determined by comparing the Bayesian information criterion (BIC) and Akaike information criterion (AIC). When competing models showed very similar AIC values, the more parsimonious model was retained when it was also supported by a lower BIC. Importantly, this clustering approach was not intended for inferential statistical testing, but rather to provide an initial framework for characterizing pattern structure in a limited dataset. Because all bite marks were obtained from a single pectoral fin of one individual within a single documented interaction context, individual bite marks were not treated as independent biological replicates.

## Results

### Behavioral observations

A biting event by a male whale shark directed toward a female was recorded using surveillance cameras on May 12, 2020. Before the biting event, three types of approach behaviors were observed: (1) the male positioned himself above the dorsal surface of the female, aligned his ventral side along her flank, and moved toward the pectoral fin (Fig. 2A); (2) the male approached laterally and attempted to bring his mouth close to the pectoral fin (Fig. 2B); or (3) the male approached from behind and moved ventrally toward the pectoral fin (Fig. 2C).

**Figure 2:**
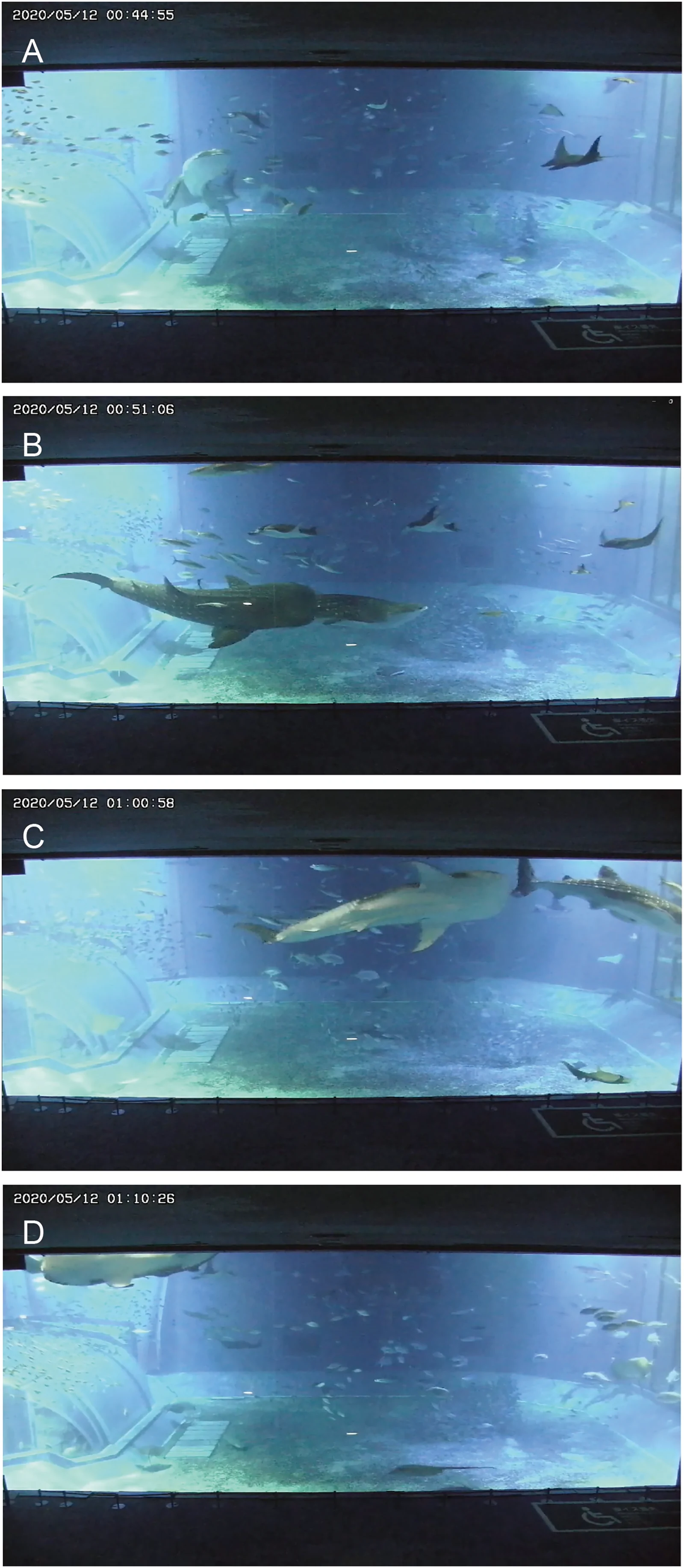
Approaches of the male whale shark toward the female. (A) From the dorsal side; (B) from the lateral side toward the pectoral fin; (C) from the posterior side; and (D) grasping of the pectoral fin.

This procedure was repeated multiple times. At 0110 h, following approach type (2), the male bit the distal region of the right pectoral fin of the female (Fig. 2D; Videos S1 and S2). This was the only pectoral-fin biting event observed during continuous behavioral monitoring, and no additional biting events involving the affected pectoral fin were recorded before or after this event. The duration of the biting event could not be determined owing to the blind spots of the camera view and obstruction by the tank wall. Video footage further showed that the male and female swam side by side for approximately 30 s following the bite. No insertion of the clasper into the female cloaca was observed, and subsequent examinations revealed no abrasions or abnormalities around the female cloacal region or the male claspers.

### Healing of bite marks

The healing process of the pectoral fin bite marks was documented intermittently from May 12, 2020, until the death of the female on June 17, 2021. Immediately after the biting event, the injury consisted of open wounds with exposed subcutaneous tissue and superficial epidermal abrasion (Fig. 3A). Approximately 4 weeks after the injury, the open wounds were closed (Fig. 3B). Following wound closure, progressive dark pigmentation developed at the sites of injury. Approximately 12 weeks post-injury, pigmentation became more pronounced and regeneration of dermal denticles was observed (Fig. 3C). This pigmentation persisted throughout the monitoring period and remained clearly visible at the time of death of the female, approximately 13 months after the biting event (Fig. 3D). Microscopic examination of the skin samples collected during necropsy confirmed melanin deposition in the regenerated dermal denticles within the scarred regions (Fig. 3E).

**Figure 3:**
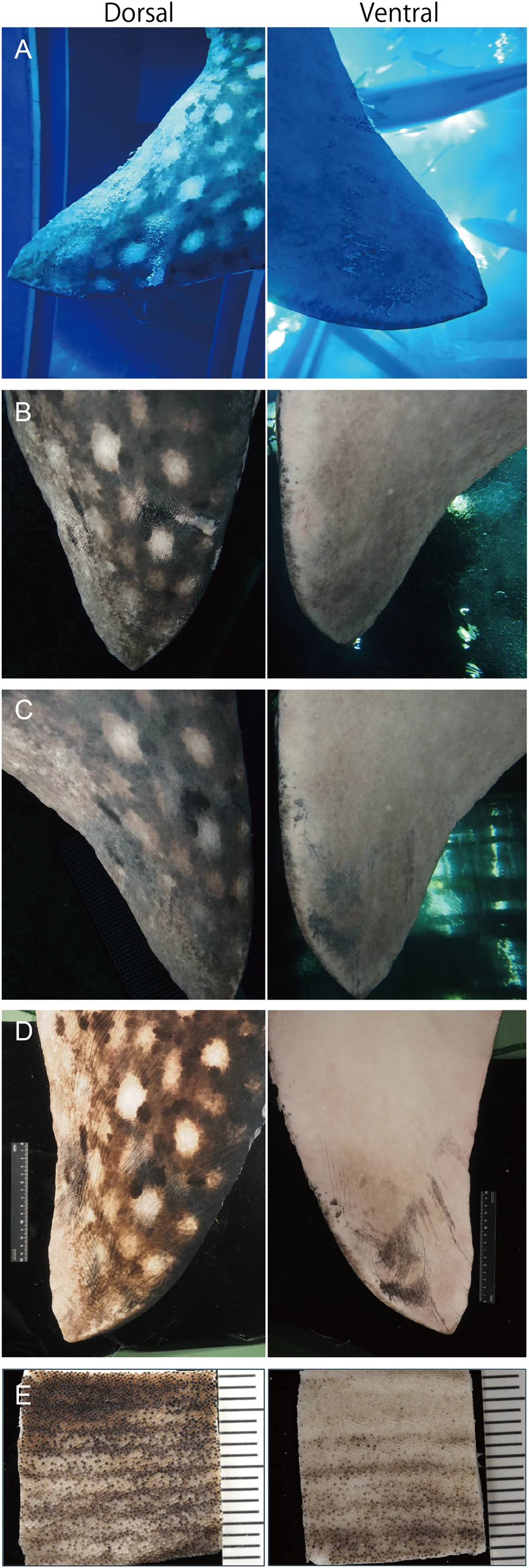
Healing process of bite marks on the right pectoral fin of a female whale shark. (A) Fresh wound immediately after the bite by the male on May 12, 2020; (B) scar approximately 4 weeks later on June 6, 2020; (C) darkened pigmentation observed approximately 12 weeks later on August 5, 2020; (D) bite marks recorded post-mortem on June 22, 2021; and (E) close-up view of part of the wound (ca. 2 cm × 2 cm).

### Verification of bite mark placement

A total of 233 dorsal and 290 ventral bite marks were identified using Bézier curves traced from the pectoral fin surfaces. PCA applied to each curve revealed that PC1 explained 99.86% of the total variance, indicating that the variation in scar geometry was dominated by a single directional component (Table 1). Therefore, the PC1 vector is used to represent the primary orientation of each bite mark.

**Table 1:** Principal component contributions and explained variance for Bézier curve direction data.

PC	Eigenvalue	Explained variance (%)	Cumulative variance (%)
PC1	85.41	99.86	99.86
PC2	0.12	0.14	100.00

The distribution of orientation angles derived from PC1 exhibited clear multimodality. Gaussian mixture model clustering applied independently to dorsal and ventral datasets supported a three-cluster structure for both surfaces. For the ventral dataset, the four-component model had a marginally lower AIC than the three-component model (2,656 *vs*. 2,657), whereas BIC favored the three-component model; therefore, the more parsimonious three-component solution was retained according to the model-selection criterion defined above. The individual cluster assignments for dorsal and ventral bite marks based on orientation angles are provided (Tables S1 and S2). For the selected three-component models, the BIC and AIC values were 2,150 and 2,122 for dorsal scars and 2,686 and 2,657 for ventral scars, respectively (Table 2).

**Table 2:** Model selection results of Gaussian Mixture Model (GMM) clustering for dorsal and ventral bite marks based on Bayesian Information Criterion (BIC) and Akaike Information Criterion (AIC).

	BIC		AIC	
Number of components	Dorsal	Ventral	Dorsal	Ventral
1	2,186	2,756	2,179	2,748
2	2,190	2,731	2,173	2,713
3	2,150	2,686	2,122	2,657*
4	2,166	2,697	2,128	2,656
5	2,182	2,712	2,133	2,661
6	2,184	2,720	2,125	2,657
7	2,191	2,737	2,122	2,663
8	2,208	2,747	2,128	2,663
9	2,217	2,764	2,128	2,668

**Note:**

* Although the minimum AIC value for the ventral side was obtained with four components (AIC = 2,656), a three-component model (AIC = 2,657) was selected as the optimal solution considering overall model parsimony and consistency with BIC.

Cluster-specific orientation distributions showed distinct ranges with limited overlaps (Fig. 4), suggesting that bite marks were not randomly distributed in orientation space. Instead, scars were grouped into discrete directional patterns that may reflect consistent biting positions or repeated jaw placement during interaction events.

**Figure 4:**
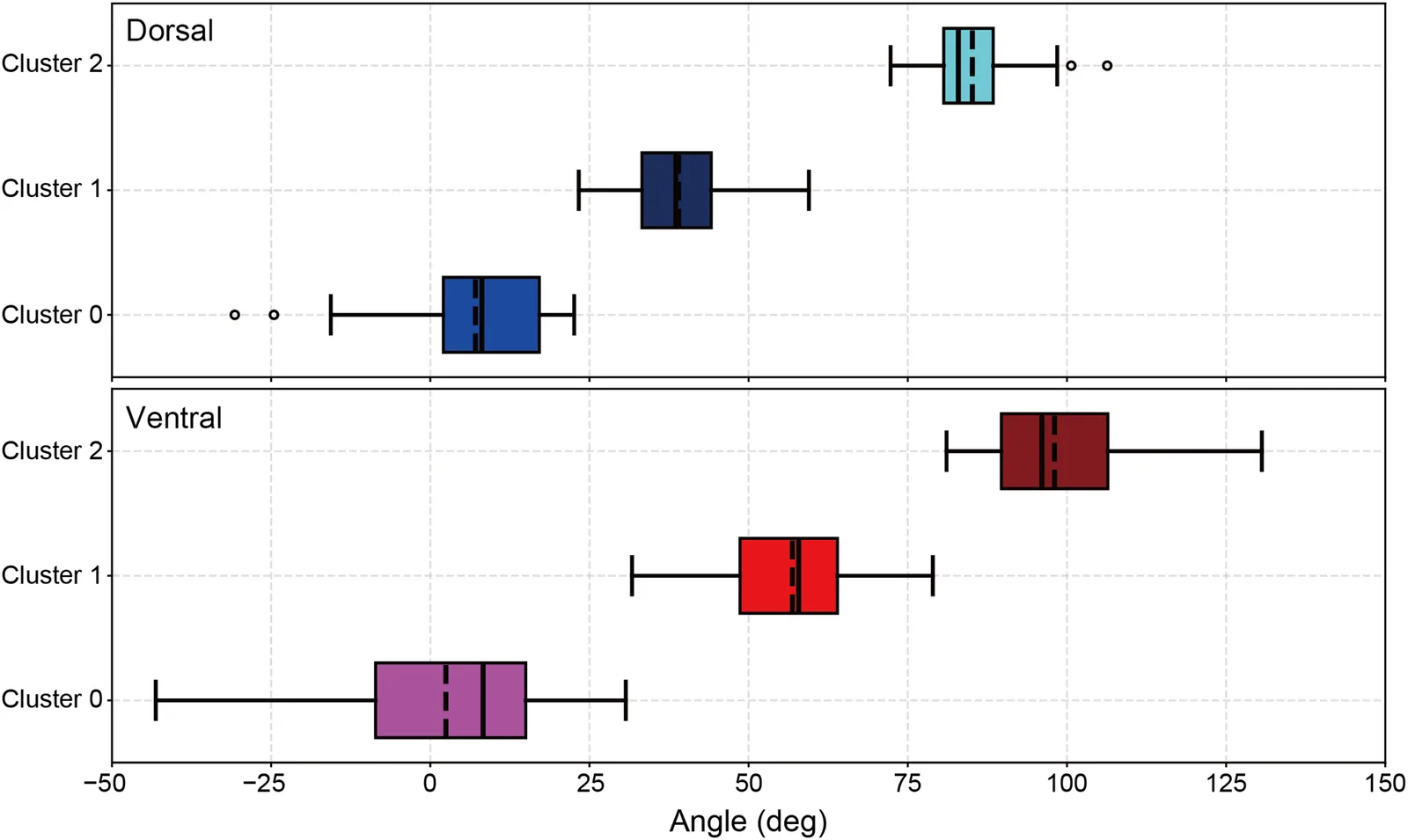
Boxplots of bitemark orientation angles for each cluster identified using a Gaussian mixture model. The upper panel shows the dorsal surface, and the lower panel shows the ventral surface. Each box represents the interquartile range (IQR), with the central line indicating the median and the dotted line showing the mean orientation angle. Whiskers extend to 1.5 × IQR, and circles denote the outliers.

The mean orientation angles of the three dorsal clusters were 7.10, 39.03, and 85.13°, whereas those of the ventral clusters were approximately 2.45, 56.89, and 98.03° (Table 3).

**Table 3:** Summary statistics of bitemark orientation clusters identified by Gaussian Mixture Model (GMM) analysis.

Surface	Cluster ID	Count of scars	Mean orientation (°)	Standard deviation	Variance
Dorsal	0	72	7.10	11.67	136.29
	1	131	39.03	8.57	73.44
	2	30	85.13	7.72	59.57
Ventral	0	41	2.45	16.24	263.79
	1	213	56.89	10.29	105.85
	2	36	98.03	12.11	146.68

Taken together, these results suggest that the multiple bite marks produced during the single documented interaction event may reflect repeated or repositioned jaw contact. These comparisons are descriptive and are intended to characterize geometric pattern structure rather than to test statistical significance.

## Discussion

This study represents the first quantitative documentation of pectoral fin bite marks inflicted by a male whale shark on a female, along with a long-term assessment of the healing trajectory in aquaria. We combined direct behavioral observations, geometric analyses of bite marks, and post-mortem tissue examinations to present rare empirical evidence linking male–female interaction behavior to externally observable scars in this species. These findings provide new insights into the interaction patterns of whale sharks, a topic that has remained largely inaccessible due to the rarity of direct observations in the wild.

Surveillance camera footage confirmed that the male repeatedly approached the female and ultimately bit the distal region of the pectoral fin. Although no clasper insertion was observed and copulation was not confirmed, the biting behavior documented in this study is broadly consistent with pre-copulatory or courtship-related interactions described in many elasmobranch species, in which males grasp the pectoral fins or flanks of females during mating attempts (Pratt & Carrier, 2001; Tricas & Le Feuvre, 1985; Santos et al., 2022). However, it is important to note that such interactions do not always lead to successful copulation, and incomplete or unsuccessful attempts have been reported in several species (Ritter & Amin, 2018). Therefore, the observed biting event may represent a pre-copulatory interaction or mating attempt, but it may also reflect an incomplete or atypical interaction that did not progress to copulation. Given the absence of genital contact or associated abrasions, the behavior should be interpreted cautiously, and we refrain from inferring successful mating from this observation. Nevertheless, such biting and holding behaviors themselves are consistent with those documented in other elasmobranch species, including blacktip reef sharks, *Carcharhinus melanopterus*, where males grasp the pectoral fin during copulation (McCauley, Papastamatiou & Young, 2010), and zebra sharks, which exhibit prolonged pursuit accompanied by repeated biting of the pectoral, dorsal, and caudal fins (Watson & Janse, 2017). Therefore, the spatial distribution and directional consistency of bite marks observed in the present study are compatible with the holding behaviors observed in other elasmobranch taxa.

Directional analyses of the bite marks revealed that scars on both the dorsal and ventral surfaces of the pectoral fin were organized into three principal orientation clusters. Because no additional pectoral-fin biting events were observed during continuous behavioral monitoring, the analyzed bite marks were attributed to the single documented interaction event. Within this context, the three orientation clusters may reflect variation in jaw orientation or repositioning during the event, although the clustering analysis alone cannot determine the specific biting sequence or jaw movements that generated these patterns. Previous studies have noted that in elasmobranchs, particularly when copulation is unsuccessful, males may repeatedly attempt to secure a grip on females (Ritter & Amin, 2018). Therefore, the number of bite marks observed on a female should not be interpreted as a direct proxy for the number of interaction events or encounters.

In the present case, a clear pectoral fin bite was recorded; however, there was no evidence of clasper insertion or genital abrasion. Therefore, bite marks should be interpreted primarily as indicators of male–female encounters rather than as confirmation of mating or reproductive success. This interpretation is consistent with findings from other large sharks, such as tiger sharks, *Galeocerdo cuvier*, in which bite marks have been documented without direct evidence of fertilization (Rangel et al., 2023).

The healing process of bite marks in the whale shark exhibited two distinct temporal phases. The open wounds closed within approximately 4 weeks, after which dermal denticle regeneration and progressive melanization occurred. Notably, the pigmentation persisted for more than 13 months, and microscopic examination confirmed the deposition of melanin in the regenerated denticles. Wound closure rates were broadly comparable to those reported in other elasmobranchs, and the long persistence of pigmentation contrasts with observations in species, such as the blacktip reef shark, the white shark, and the sand tiger shark, in which scars often fade within weeks to months (Chin, Mourier & Rummer, 2015; Towner, Smale & Jewell, 2012; Wyffels et al., 2025). The prolonged visibility of scars in whale sharks may be related to species-specific skin characteristics, including thick dermis and dense dermal denticles (Compagno, 2001).

These healing characteristics have important implications for the interpretation of bite marks in free-ranging whale sharks. In the present individual, wound closure occurred within approximately 1 month, providing a preliminary reference for the temporal interpretation of recent bite marks. However, healing rates may vary among individuals according to age, physiological condition, environmental conditions, wound severity, and other factors. Therefore, application of this timeframe to free-ranging whale sharks should be interpreted with caution until additional observations from other individuals and conditions become available. Conversely, the persistence of melanized scars for more than 13 months demonstrates that healed bite marks can remain externally visible for an extended period in this individual. Whether scar appearance can reliably be used to infer the timing or number of previous male–female encounters in free-ranging whale sharks requires validation across additional individuals. At aggregation sites where reproductive behavior or the presence of putative gravid females has been reported, such as St. Helena (Perry et al., 2020) and Darwin Island (Acuña-Marrero et al., 2014; Hearn et al., 2016), a systematic documentation of scar frequency and healing stages may improve the temporal resolution in identifying mating periods and potential breeding habitats.

Although the bite marks observed in the present study were found only on the right pectoral fin, male whale sharks can use either claspers during their courtship or mating behavior (Matsumoto et al., 2019). In closely related orectolobiform sharks, including zebra sharks and Tawny nurse sharks (Vélez-Zuazo & Agnarsson, 2011), bites are frequently observed on both pectoral fins during the breeding season and often involve multiple males (R Matsumoto & K Murakumo, 2026, personal communication). These observations highlight the importance of examining both pectoral fins when assessing bite marks in field studies to avoid underestimating encounter frequency.

This study has several limitations. First, it is based on a single case involving one immature female and one mature male whale shark. Comprehensive documentation of courtship behavior, bite mark formation, and subsequent healing within the same individuals would require long-term, continuous monitoring supported by reliable individual identification. Such conditions are rarely attainable in the wild, and only a limited number of facilities worldwide are capable of maintaining and observing large migratory elasmobranchs over extended periods. Consequently, caution is warranted when generalizing the present findings. Second, because the observations were conducted under managed conditions, encounter frequencies and spatial dynamics among individuals may differ from those in natural environments. Although the biting behaviors observed here are consistent with courtship and mating-attempt behaviors reported in wild elasmobranchs, the potential influence of captive-specific conditions on the frequency, duration, or repetition of biting events cannot be fully excluded. Bite marks primarily reflect male–female encounters, and their use as proxies for mating or reproductive success therefore requires cautious interpretation in conjunction with complementary behavioral and morphological indicators, such as abrasions around the cloaca associated with clasper insertion.

Despite these limitations, this study provides the first systematic and quantitative analysis of pectoral-fin bite marks in whale sharks, including explicit characterization of wound-healing timelines. These findings provide a preliminary basis for interpreting interaction-related scars in wild individuals. Future studies integrating underwater observations and citizen-science datasets from aggregation sites with AI-based automated scar detection (Holmberg, Norman & Arzoumanian, 2009; Norman et al., 2017), together with physiological indicators such as hormone profiles (Matsumoto et al., 2023), are expected to further strengthen reproductive monitoring approaches across both *in situ* and *ex situ* contexts.

Overall, this study provides a preliminary framework for evaluating pectoral fin bite marks as potential indicators of male–female interaction history in whale sharks. By integrating *ex situ* behavioral observations with quantitative scar analyses, we provide a methodological basis for linking aquarium-based observations with future field studies. Validation across additional individuals and, ideally, *in situ* observations will be required before scar characteristics can be reliably used to infer encounter timing or interaction history in free-ranging whale sharks.

## Supplemental Information

10.7717/peerj.21809/supp-1 Supplemental Information 1 Photographs of the study animals. (A) Immature female whale shark (*Rhincodon typus*; 8.0 m total length [TL]) monitored for pectoral fin bite wound formation and subsequent healing processes. (B) Male whale shark (*R. typus*; 8.8 m TL) observed performing pectoral fin biting behavior during mating attempts.

10.7717/peerj.21809/supp-2 Supplemental Information 2 Bitemarks dorsal without outline: SVG data of bite marks on the dorsal side of the right pectoral fin. Raw data consisting of SVG files representing individual bite marks observed on the dorsal surface of the right pectoral fin of a female whale shark (*Rhincodon typus*). Each SVG file contains a vectorized Bézier-curve representation of a single bite mark, digitized from photographic images using Adobe Illustrator, without inclusion of the pectoral fin outline. These raw geometric data were used as the input for subsequent quantitative analyses, including angle measurements, principal component analysis (PCA), and Gaussian mixture model (GMM) clustering.

10.7717/peerj.21809/supp-3 Supplemental Information 3 Surveillance camera footage showing a male whale shark approaching and biting the distal region of the right pectoral fin of a female. Videos were recorded using a ceiling-mounted surveillance camera installed at the front of the tank (WV-SC588; Panasonic Corp., Osaka, Japan). The camera captured the primary sequence of the biting event and provided the reference time used in the behavioral analysis.

10.7717/peerj.21809/supp-4 Supplemental Information 4 Bitemarks ventral without outline: SVG data of bite marks on the dorsal side of the right pectoral fin. Raw data consisting of SVG files representing individual bite marks observed on the ventral surface of the right pectoral fin of a female whale shark (*Rhincodon typus*). Each SVG file contains a vectorized Bézier-curve representation of a single bite mark, digitized from photographic images using Adobe Illustrator, without inclusion of the pectoral fin outline. These raw geometric data were used as the input for subsequent quantitative analyses, including angle measurements, principal component analysis (PCA), and Gaussian mixture model (GMM) clustering.

10.7717/peerj.21809/supp-5 Supplemental Information 5 GMM results of dorsal bite marks. Gaussian mixture model (GMM) clustering results based on angular measurements of bite marks observed on the dorsal side of the right pectoral fin of a female whale shark (*Rhincodon typus*). The dataset includes the measured bite mark angles (in degrees) and the corresponding cluster assignments generated by the GMM analysis. Each row represents a single bite mark. Cluster numbers indicate relative groupings within the dataset and do not imply any ordinal, temporal, or biological ranking. These data represent processed analytical results derived from the raw SVG-based Bézier curve data.

10.7717/peerj.21809/supp-6 Supplemental Information 6 Bézier control point coordinates for pectoral fin bite marks. Raw data consisting of two-dimensional coordinate points used as the original input for constructing Bézier-curve representations of bite marks on the pectoral fins of a female whale shark (*Rhincodon typus*). The dataset includes x–y coordinate values of digitized points defining individual bite mark paths, with each path identified by a path index and classified by surface (dorsal or ventral). These coordinates were manually extracted from photographic images using Adobe Illustrator and represent the raw point data prior to Bézier curve fitting and SVG generation. This dataset served as the foundational raw data for subsequent geometric analyses of bite mark shape and orientation.

10.7717/peerj.21809/supp-7 Supplemental Information 7 GMM results of ventral bite marks. Gaussian mixture model (GMM) clustering results based on angular measurements of bite marks observed on the ventral side of the right pectoral fin of a female whale shark (*Rhincodon typus*). The dataset includes the measured bite mark angles (in degrees) and the corresponding cluster assignments generated by the GMM analysis. Each row represents a single bite mark. Cluster numbers indicate relative groupings within the dataset and do not imply any ordinal, temporal, or biological ranking. These data represent processed analytical results derived from the raw SVG-based Bézier curve data.

10.7717/peerj.21809/supp-8 Supplemental Information 8 Simultaneous auxiliary camera footage of the same biting event shown in Video S1, recorded from the opposite side of the tank. To compensate for the blind spots of the primary camera, an additional camera was installed in the observation window on the opposite side of the tank (WTW-P27HT-64GB; Wireless Tsukamoto Co. Ltd., Mie, Japan). Although this video corresponds to the same event as shown in Video S1, the timestamp displayed in Video S2 was delayed by more than 1 min relative to the primary camera; therefore, the timing shown in Video S1 was used as an accurate reference.

## Additional Information and Declarations

### Data Availability

The following information was supplied regarding data availability:

The data is available in the Supplemental Files.
